# Analysis of Various Factors Associated With Opioid Dose Escalation in Patients With Cancer Pain

**DOI:** 10.7759/cureus.25266

**Published:** 2022-05-24

**Authors:** Ryo Sakamoto, Atsuko Koyama

**Affiliations:** 1 Department of Psychosomatic Medicine, Kindai University Faculty of Medicine, Osakasayama, JPN

**Keywords:** pain, cancer, alcohol consumption, polypharmacy, opioid dose escalation

## Abstract

Introduction

Pain is one of the most important symptoms in terms of prevalence and a major cause of distress in patients with cancer. Therefore, this study aimed to analyze and identify the factors that influence the worsening of pain in patients with cancer necessitating opioid dose escalation.

Methods

The study was conducted in a single center. This study is a retrospective cohort study of 390 adult cancer patients. The primary endpoint was dose escalation for strong opioids. Adjusted odds ratios (aORs) and their 95% confidence intervals (CIs) were calculated using a logistic regression model to evaluate the relationships of factors with opioid dose escalation for cancer pain.

Results

Polypharmacy was associated with opioid dose escalation (aOR = 2.54, 95% CI = 1.486-4.370, p = 0.001). Conversely, alcohol consumption was associated with a reduced need for dose escalation (aOR = 0.60, 95% CI = 0.376-0.985, p = 0.043).

Conclusion

The results of this study indicate that moderate alcohol consumption does not reduce the efficacy of opioids in patients with cancer pain. Meanwhile, patients receiving polypharmacy may be able to more rapidly alleviate their pain via early opioid dose modification.

## Introduction

The incidence and incidence rate of cancer patients is increasing from year to year [1]. Globally, it is estimated that 18.1 million people will develop cancer, and 9.6 million died of cancer in 2018. In Japan, 1.01 million people were diagnosed with cancer in 2020, and 379,400 people died of cancer [2]. Although the symptoms of cancer vary widely, cancer pain is one of the most frequent and severe symptoms experienced by patients requiring palliative care [3]. Cancer pain is present in 39.3% of patients after curative treatment, 55% of patients during anticancer treatment, 66.4% of patients with advanced, metastatic, or terminal disease, and 50.7% of all patients with cancer. Moderate to severe pain has also been reported in 38% of patients with cancer [4,5]. Morphine is recommended as the first-line treatment for controlling moderate to severe cancer pain [6]. According to the World Health Organization guidelines for the pharmacological treatment of cancer pain published in 2019, cancer pain is classified by neural mechanisms as either nociceptive or neuropathic. However, not all types of pain in patients with cancer are solely related to the tumor, and thus, they cannot automatically be defined as cancer pain. A large prospective study conducted on patients with cancer illustrated that approximately 17% of the pain perceived by the patients was caused by antitumor treatments and approximately 10% was attributable to other etiologies unrelated to cancer [7]. Among patients with cancer pain, more than 40% experience inadequate pain relief and remain potentially undertreated [8]. Cancer pain can potentially alter the quality of life (QOL) of patients [9], and it has physical, psychological, and emotional effects on daily and social life [10]. In patients with cancer pain, it is critical to identify whether the perceived pain is caused by treatment or other factors in order to provide necessary treatment. Exploring the factors that contribute to worsening cancer pain can facilitate the development of countermeasures to prevent future worsening of pain. We believe that if pain can be controlled at an early stage, patients’ motivation to continue treatment and their QOL can be improved. Therefore, this study investigated the factors resulting in opioid dose modification because of cancer pain at our hospital.

## Materials and methods

Study design

This study was a retrospective investigation of patients with cancer pain who were started on strong opioids and required dose escalation within one month. Strong opioids are defined by the WHO Cancer pain relief. Eligible patients had histories of surgery, pharmacotherapy, radiotherapy, and palliative treatment.

Participants

From October 2017 to September 2020, we conducted a retrospective study using the electronic medical records of eligible cancer pain patients who visited a hospital affiliated with Kindai University and were prescribed strong opioids for the first time at our hospital. Patients with missing data in their medical records were excluded. After excluding three patients with missing study items in the electronic medical record, 390 patients were included in the study.

Demographic information

Background information included age, sex, and comorbidities; bone metastasis; performance status; the receipt of analgesics other than opioids; the use of psychotropic drugs, chemotherapy, radiotherapy, or polypharmacy; residential status; area of residence; smoking; and alcohol history. Polypharmacy was defined as the concurrent use of five or more drugs [11], excluding cancer drugs, because many patients were undergoing active cancer treatment. This study was approved by the Kindai University Hospital Clinical Research Ethics Review Committee (approval number 2020-268). The research process and the preparation of this paper were guided by the Declaration of Helsinki’s ethical principles for medical research involving human subjects.

Endpoints

The primary endpoint was the dose escalation of strong opioids. The analyzed strong opioids were morphine, oxycodone, hydromorphone, tapentadol, fentanyl, and methadone. The primary study endpoint was baseline opioid dose escalation within one month. Dose titration was defined as an addition to the originally prescribed baseline daily opioid dose. The decision to increase the opioid dose was made by the attending physician based on patient complaints. The approximate range of opioid escalation was 30%-50% per dose. In the study’s definition of increased opioid dosage, the number of titrations within a period of time was irrelevant. The evaluation period was within one month. The reason for this is that follow-up should occur as early as possible to assess the need for an opioid dosage increase [12].

Statistical analyses

Each drug was determined using Microsoft Excel version 16.0 (Microsoft Corp., Redmond, WA, USA). A comparison of the opioid dose escalation group and no opioid dose escalation group was performed using Welch’s t-test. To evaluate the relationships between variables and opioid dose escalation for cancer pain, adjusted odds ratios (aORs) and their 95% confidence intervals (CIs) were calculated using a logistic regression model. Multiple logistic regression was performed with adjustment for all potential confounders listed in the endpoints. All statistical analyses were performed using SPSS version 25 (SPSS, Inc., Chicago, IL, USA).

## Results

The characteristics of the target patients are presented in Table 1. The included patients had a mean age of 66.2 ± 11.8 years, and 62% of the subjects were male. The most common type of cancer was lung cancer (23%), followed by head and neck cancer (15%) and colorectal cancer (9%). Of the patients, 31% had bone metastases from cancer, 72% had a performance status of 0-2, 49% were receiving chemotherapy, and 13% were receiving radiotherapy. Meanwhile, 42% of the patients required opioid dose escalation.

**Table 1:** Patient characteristics

	n (%)
Age, mean (standard deviation; range)	66.2 (11.8; 27-93)
Sex (%)	
Men	243 (62.3)
Women	147 (37.7)
Cancer	
Primary site (%)	
Lung	92 (23.5)
Head and neck	59 (15.1)
Duodenum, colon, rectum	36 (9.2)
Pancreatic	34 (8.7)
Esophagus	26 (6.6)
Breast	22 (5.6)
Stomach	19 (4.8)
Hepatobiliary	19 (4.8)
Urinary system	19 (4.8)
Unknown primary	12 (3)
Uterus and ovaries	10 (2.5)
Blood	6 (1.5)
Thyroid	6 (1.5)
Malignant pleural mesothelioma	6 (1.5)
Other	24 (6.1)
Bone metastasis	
Yes	123 (31.5)
No	267 (68.5)
Performance status (%)	
0-2	282 (72.3)
3-4	108 (27.7)
Opioid base-up (within one month)	
Yes	167 (42.8)
No	223 (57.2)
Chemotherapy	
Yes	192 (49.2)
No	198 (50.8)
Radiotherapy	
Yes	52 (13.3)
No	338 (86.7)

The comorbidities, medications, and social backgrounds of the patients are presented in Table 2. Comorbidities were found in 70.5% of the patients, and 70.2% of the patients received polypharmacy. Analgesics other than opioids were administered to 78.4% of the patients, and 28.9% of the patients received psychotropic drugs. In total, 15.6% of the patients lived alone, and 74.8% of the patients resided in municipalities surrounding Osakasayama City. Smoking and drinking were recorded for 21% and 38.2% of the patients, respectively.

**Table 2:** Patient’s drug and social background

	n (%)
Comorbidity	
Yes	275 (70.5)
No	115 (29.5)
Polypharmacy	
Yes	274 (70.2)
No	116 (29.8)
Analgesics (non-opioid)	
Yes	306 (78.4)
No	84 (21.6)
Psychotropic	
Yes	113 (28.9)
No	277 (71.1)
Household	
Solitary life	61 (15.6)
Gregariousness	329 (84.4)
Distance to the hospital	
Surrounding municipalities	292 (74.8)
More remote than nearby	98(25.2)
Smoking	
Yes	82 (21)
No	308 (79)
Drinking	
Yes	149 (38.2)
No	241 (61.8)

The comparison of drug doses between the groups with and without increasing opioid doses is shown in Table 3. Oral morphine equivalent daily dose (OMEDD) was converted for each opioid using the equivalence conversion table as a reference [13-15]. The results of the t-test showed that the differences in the mean daily doses of OMEDD (p = 0.0033), morphine (po) (p = 0.0002), and hydromorphone (po) (p = 0.0027) were significant.

**Table 3:** Comparison of drug dose between the opioid dose escalation group and the no opioid dose escalation group Drugs are shown as means and standard deviations (±SD). SD, standard deviation; OMEDD, oral morphine equivalent daily dose; po, oral; iv, intravenous; td, transdermal; ns, not significant

Drugs (mg)	Opioid dose escalation group (n = 167)	No opioid dose escalation group (n = 223)	p-value
OMEDD	28.525 (48.499)	34.556 (63.561)	0.033
Each strong opioid (daily dose)			
Morphine (po)	23.333 (4.714)	32.857 (12.777)	0.002
Morphine (iv)	None	25	ns
Oxycodone (po)	29.150 (28.095)	27.733 (37.249)	ns
Oxycodone (iv)	22.916 (12.112)	17.5	ns
Hydromorphone (po)	4.00 (11.25)	7.866 (11.086)	0.027
Fentanyl (td)	1.545 (1.157)	1.804 (1.324)	ns
Fentanyl (iv)	0.12	1.200 (1.080)	ns
Tapentadol	183.333 (62.360)	135.714 (58.028)	ns
Methadone	45	None	ns
Each analgesic (daily dose)			
NSAIDs (po)			
Loxoprofen	172.881 (27.249)	170.769 (34.072)	ns
Celecoxib	322.222 (97.499)	254.544 (89.072)	ns
Diclofenac sodium	37.500 (12.500)	50.000 (22.360)	ns
Etodolac	400	400	ns
Naproxen	200	400.000 (167.332)	ns
NSAIDs (iv)			
Flurbiprofen axetil	150	150	ns
Acetaminophen	2218.220 (843.781)	2098.928 (808.100)	ns
Pregabalin	151.250 (108.245)	147.727 (88.840)	ns
Mirogabalin	13.750 (9.601)	14.000 (8.602)	ns
Duloxetine	44.000 (14.966)	33.333 (9.423)	ns
Amitriptyline hydrochloride (po)	25	None	ns
Corticosteroids (po)			
Betamethasone	2	1.833 (0.372)	ns
Dexamethasone	4.250 (3.750)	1	ns
Prednisolone	16.888 (13.135)	7.000 (2.449)	ns
Corticosteroids (iv)			
Betamethasone	6 (2.000)	6.181 (1.991)	ns
Dexamethasone	7.333 (3.023)	7.228 (2.414)	ns

Table 4 presents the results of the regression analysis for patients requiring dose escalation. Polypharmacy (aOR = 2.54, 95% CI = 1.486-4.370, p = 0.001) was associated with opioid dose escalation. Conversely, alcohol consumption was associated with a reduced need for dose modification (aOR = 0.60, 95% CI = 0.376-0.985, p = 0.043). No other factors were associated with opioid dose escalation.

**Table 4:** Odds ratios and 95% confidence intervals for factors influencing opioid dose escalation for cancer pain OR, odds ratio; CI, confidence interval

	Univariate analysis			Multivariate analysis		
		95% CI			95% CI	
Variable	OR	Lower-upper	p-value	OR	Lower-upper	p-value
Age	0.99	0.980-1.014	0.746	0.98	0.967-1.006	0.163
Gender						
Women	Reference			Reference		
Men	0.91	0.462-1.059	0.091	0.87	0.569-1.483	0.729
Bone metastasis						
No metastasis	Reference			Reference		
Metastasis	1.73	1.126-2.675	0.013	1.32	0.827-2.113	0.243
PS						
0-2	Reference			Reference		
3-4	1.76	1.124-2.761	0.013	0.63	0.378-1.077	0.092
Chemotherapy						
No dosing	Reference			Reference		
Dosing	0.74	0.497-1.115	0.152	0.92	0.575-1.471	0.727
Radiotherapy						
Not in progress	Reference			Reference		
In progress	0.52	0.275-0.991	0.047	0.59	0.298-1.196	0.146
Comorbidity						
None	Reference			Reference		
Yes	1.15	0.738-1.797	0.533	1.17	0.703-1.966	0.538
Polypharmacy						
None	Reference			Reference		
Yes	2.28	1.431-3.632	0.001	2.54	1.486-4.370	0.001
Analgesics (non-opioid)						
None	Reference			Reference		
Yes	1.98	1.179-3.345	0.010	1.63	0.927-2.893	0.089
Psychotropic						
None	Reference			Reference		
Yes	0.96	0.618-1.498	0.863	0.66	0.402-1.090	0.105
Household						
Gregariousness	Reference			Reference		
Solitary life	1.56	0.902-2.702	0.112	1.62	0.922-2.867	0.093
Distance to the hospital						
Surrounding municipalities	Reference			Reference		
More remote than nearby	0.80	0.503-1.285	0.036	1.40	0.849-2.322	0.186
Smoking						
No	Reference			Reference		
Yes	0.59	0.355-0.991	0.046	0.57	0.325-1.007	0.053
Drinking						
No	Reference			Reference		
Yes	0.54	0.354-0.829	0.005	0.60	0.376-0.985	0.043

## Discussion

The first important finding of this study was that drinking habits may not increase the need for opioid dose modification in patients with cancer pain. Previous studies have reported that chronic pain often results in the concurrent use of alcohol and opioids and that excessive alcohol consumption has a negative impact on pain [16]. The periaqueductal gray (PAG), also known as the pain circuit, plays a central role in nociception, and it has been implicated in the pathogenesis of anticipated pain and perceived pain [17,18]. PAG also influences pain sensitivity associated with problematic alcohol consumption and alcohol-induced changes in brain mechanisms that underpin PAG-mediated stress responses and pain transmission [19-21]. Excessive alcohol consumption is associated with pain, often through alcohol-induced changes in brain mechanisms that support PAG-mediated stress responses and pain transmission [22]. Excessive alcohol intake has negative effects on pain; however, it has also been suggested that PAG, which is involved in the pain circuitry, and the medial orbitofrontal cortex, which is involved in the reward circuitry, act antagonistically to modulate alcohol expectancy and control drinking behavior [23]. In fact, it has been reported that patients with chronic non-cancer pain are less likely to drink alcohol, and alcohol consumption is further reduced in opioid users [24]. Thus, it is possible that drinking habits that do not result in excessive alcohol consumption, as indicated by our findings, may have some beneficial effects on patient QOL by stimulating the reward system without exerting a negative effect on pain, thereby slowing the worsening of pain. In addition, the study results suggested that polypharmacy may affect the need for opioid dose escalation for cancer pain. Polypharmacy is common among prefrail and frail adults [25]. Patients with cancer experience fatigue associated with cancer progression, treatment, and other factors, and this fatigue can exacerbate pain [23]. Moreover, polypharmacy has also been found to be associated with poor health-related QOL [26]. Patients’ subjective health conditions range from disorders of mental health and vitality to pain [27]. Thus, a decrease in health-related QOL may be associated with worsening pain. These factors may have also led to an increase in the dose of strong opioids.

Five limitations of this study must be mentioned. First, all patients were recruited from a single institution. According to Japanese national cancer incidence data, men are more likely to develop stomach cancer, trachea, bronchus, and lung (TBL) cancer, and colorectal cancer, whereas women are more likely to develop breast cancer, colorectal cancer, and stomach cancer [28]. According to global cancer incidence data, men are more likely to develop skin cancer, TBL cancer, and prostate cancer, whereas women are more likely to develop non-melanoma skin cancer, breast cancer, and colorectal cancer [29]. Although these rates differ slightly from those of the study population, all of these cancer types were represented in the study, and it is unlikely that these differences affected the validity of the results. Second, the timing of opioid dose escalation was based on the date on which strong opioids were first prescribed in our hospital. Kindai University Hospital is a regional center for cancer treatment that has a system through which patients who require cancer treatment are referred or who require cancer treatment are referred for a consultation, but some patients are prescribed strong opioids prior to visiting the hospital. In other words, opioids are used in conditions where there is an acute need for them, and their use in non-acute care has not been evaluated and is a subject for future study. Third, the criteria for determining the use of polypharmacy were unclear, as it is difficult to determine whether a prescription is appropriate simply by checking patients’ medical records. It is also difficult to determine the actual level of adherence to medication. In the future, it will be necessary to use tools that enable comprehensive assessments of polypharmacy to determine both the number of medications and whether the patient is truly polyphasic [30]. Fourth, alcohol intake was only interviewed as “yes” or “no,” so it is not known how much alcohol is consumed. Finally, the results of the medical records made it difficult to classify the nature of the pain. The classification was difficult because some attending physicians did not describe nociceptive, somatic, or neuropathic pain.

## Conclusions

The results suggest that drinking habits may not increase the need for opioid dose modification and that polypharmacy may influence opioid dose escalation for cancer pain. The findings of this study may provide clues to preventing the worsening of cancer pain in the future. However, because of the limitations described in this study, further evaluation and examination of the factors are necessary in the future.
